# The long-term consequences of hybridization between the two *Daphnia *species, *D. galeata *and *D. dentifera*, in mature habitats

**DOI:** 10.1186/1471-2148-11-209

**Published:** 2011-07-15

**Authors:** Seiji Ishida, Akiko Takahashi, Noe Matsushima, Jun Yokoyama, Wataru Makino, Jotaro Urabe, Masakado Kawata

**Affiliations:** 1 Institute of International Advanced Interdisciplinary Research, Tohoku University, Aoba, Sendai, Miyagi 980-8578, Japan; 2 Division of Ecology and Evolutionary Biology, Department of Environmental Life Sciences, Graduate School of Life Sciences, Tohoku University, Aoba, Sendai, Miyagi 980-8578, Japan; 3 Department of Biology, Faculty of Science, Yamagata University,1-4-12 Kojirakawa, Yamagata, Yamagata 990-8560, Japan

## Abstract

**Background:**

Ecological specializations such as antipredator defense can reinforce morphological and distributional divergence within hybridizing species. Two hybridizing species of *Daphnia *(*D. galeata *and *D. dentifera*) are distributed in both Japan and North America; however, these populations have a longer history in Japan than in North America due to the differing impact of the last glaciation on these two regions. We tested the hypothesis that this longer coexistence in Japan would lead to extensive genetic admixture in nuclear and mitochondrial DNA whilst the distinct morphological traits and distributional patterns would be maintained.

**Results:**

The high level of correspondence among morphological traits, distribution, and mitochondrial and nuclear DNA types for the specimens with *D. dentifera *mtDNA indicated that the species distinction has been maintained. However, a discordance between mtDNA and nuclear ITS-1 types was observed for most specimens that had *D. galeata *mtDNA, consistent with the pattern seen between the two species in North America. This observation suggests nuclear introgression from *D. dentifera *into *D. galeata *without mitochondrial introgression.

**Conclusions:**

The separation of morphological traits and distribution ranges of the two hybridizing species in Japan, as well as in North America, has been maintained, despite large differences in climatic and geographical histories of these two regions. Variations in environmental factors, such as predation pressure, might affect maintenance of the distribution, although the further studies are needed to confirm this.

## Background

The factors that determine the range and distribution patterns of a species along latitudinal and altitudinal gradients have gained significant interest with the recognition that global warming has the potential to cause shifts in these patterns and change interactions among species [1-3]. The range and distribution pattern of a species can be limited by geographical barriers, interactions with other species, or failure to adapt at range margins because of lack of genetic variation, dispersal load, or stochastic extinction [4,5]. When the distribution patterns of two or more closely related species overlap, these species may hybridize and produce hybrid zones, which can affect species distribution [6]. The consequences of hybridization on the distribution patterns depend on various environmental and genetic factors, including environmental selection, dispersal ability, asymmetric hybridization, and fitness of hybrid offspring [7]. It is important to consider how these factors affect the range and distribution patterns of species under the effect of frequent hybridization.

**Figure 1:**
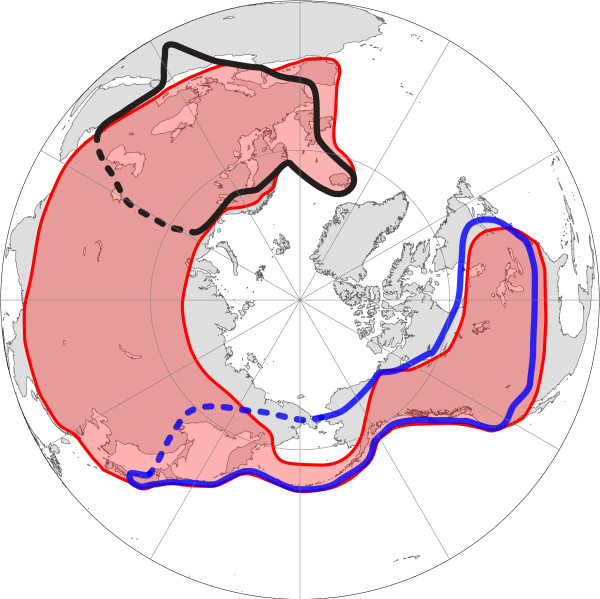
**A distribution map of *Daphnia galeata*, *D. dentifera*, and *D. Longispina***. Red-filled regions represent the distribution of *Daphnia galeata *[12]. The blue and black lines indicate the distributions of *D. dentifera *and *D. longispina *sensu Petrusek et al. [50]. A dotted line represents the putative boundaries of the distributions based on a recent phylogeographic study [21].

Members of the subgenus *Hyalodaphnia *inhabit freshwater lakes and ponds over a wide range of Holarctic regions. Hybridization between species of *Hyalodaphnia *with overlapping ranges has been observed in many dispersed locations [8]. *Daphnia galeata*, a species of *Hyalodaphnia*, has a wide Holarctic distribution [9], which overlaps with that of all the other members of *Hyalodaphnia*, except *Daphnia umbra *[10-16]. In these overlapping areas, local hybrids involving *D. galeata *are common and often more abundant in lakes than the parental taxa [8,17-19]. *Daphnia dentifera*, a vicariant taxon of *Daphnia longispina *sensu Petrusek et al. [20], also has a wide distribution in North America and Japan [21], and hybridizes with *D. galeata *in North America [15,22] (Figure 1). Nuclear loci of *D. dentifera *were introduced into most North American populations of *D. galeata *[14,15,21,23].

There are several morphological differences between *D. galeata *and *D. dentifera*, including a dark ring on the base of the distal segment of the swimming setae present in *D. dentifera *and variations in the number of post-abdominal claws [12]. The differences in phenotypic adaptations as an antipredator defense are conspicuous. Since carnivorous invertebrates (e.g., *Chaoborus *midge larvae, *Leptodora*, *Bythotreps, Cyclops*) and gape-limited young-of-the-year (YOY) fish selectively prey upon daphniids with a smaller body size, individuals with a larger body size or with other defensive attributes are less vulnerable to these predators. In response to chemical cues from these predators, *D. galeata *often elongates its head shape and forms a crest [24-29], whereas juveniles and male *D. dentifera *occasionally develop neck teeth on the dorsal margin of the helmet [12]. Both defensive traits of the crest and neck teeth are beneficial against these gape-limited predators [30,31]. However, planktivorous fish, with the exception of gape-limited fish, selectively prey upon daphniids with a larger body size [32]. Gelinas et al. [24] noted that the number of *D. galeata *possessing a crest increased during and after the season when YOY fish occurred, indicating that the head morphology of *D. galeata *is influenced at least in some ways by the presence of planktivorous fish in these habitats. *D. galeata *and *D. dentifera *are also ecologically distinct in the range of vertical habitat use within lakes and habitat distribution among lakes of different sizes [22]. Because these two species and their hybrids exhibit a stable co-existence in many lakes in North America, ecological specialization may reinforce the divergence within these two species [22].

Recent phylogeographical studies indicated that *D. galeata *and *D. dentifera *should have a longer history of coexistence in Japan than in North America [21,33]. During the last Ice Age, an ice sheet covered the northern regions of North America [34]. Ishida and Taylor [21,33] estimated that *D. galeata *and *D. dentifera *should have expanded their ranges throughout North America after the last deglaciation. On the other hand, Japan was mostly unglaciated and unoccupied by permafrost during the last Ice Age [35]. Japan should have functioned as a glacial refugium for temperate freshwater invertebrates during several glacial cycles. Indeed, one of the oldest mitochondrial clades of *D. galeata *was distributed only throughout Japan. Japanese populations of *D. dentifera *also have much a higher genetic diversity and a stronger regional structure than North American populations, indicating that Japan has provided mature habitats for the species. Consistently, recent phylogeographical studies have also indicated that Japan should be rich in regional population structures and endemic cryptic species of freshwater invertebrates [36-39].

A survey of the distribution, morphological variation, and hybridization of *D. galeata *and *D. dentifera *in Japan would provide an opportunity to test important hypotheses regarding hybridization and morphological evolution. These two species can be taxonomically distinguished by certain morphological characteristics in both Japan and North America. However, since in Japan the two species experienced different climate histories than they did in North America, and since they have a longer history of coexistence there, we hypothesised that this longer coexistence of the two species would lead to extensive genetic admixture, whereas differences in morphological traits related to ecological specialization such as an antipredation defense would be maintained. In addition, it is important to examine the environmental factors that affect the distribution and maintenance of morphological traits that characterize *D. galeata *and *D. dentifera *since this would help to predict the distribution and biodiversity of *Daphnia *species under current and future environmental changes such as global warming.

The purpose of this study is to test our hypothesis regarding the consequences of the hybridization between *D. galeata *and *D. dentifera *in Japan. We used 66 populations of *D. galeata *and *D. dentifera *distributed throughout Japan, and analyzed the relationships among geographic distribution patterns, morphological traits of head shape and body size, and genetic characteristics of mitochondrial 12SrRNA and nuclear rRNA internal transcribed spacers (ITS-1 and ITS-2). In particular, we examined (1) to what degree the morphological traits and the mitochondrial and nuclear characteristics are correlated, how these traits and characteristics are distributed in Japan; and (2) to what degree introgression occurred between these species.

## Results

### Relationship among mitochondrial genealogy, morphology, and geographic distributions

**Figure 2:**
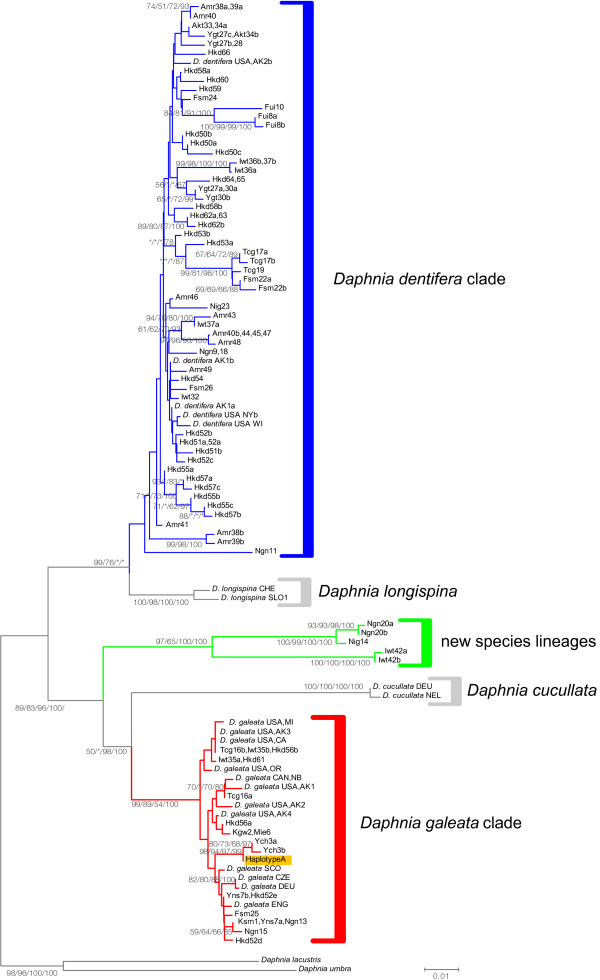
**Neighbor-joining (NJ) phylogram of mitochondrial 12SrRNA** . The four numbers on each branch indicate greater than 50% bootstrap support values for the branch as determined by NJ, maximum parsimony, and maximum likelihood methods, and a Bayesian clade credibility value of greater than 70% Asterisks indicate values with less than 50% bootstrap support values, less than 70% Bayesian support values, or no support. Population names are abbreviated [see Additional file 1]. Haplotype A in the *D. galeata *clade occurred in nine populations throughout Japan: Hrs4, Hyg5 Mie6, Ibr12, Ngn13, Fsm21, Fsm25, Ygt27, Myg29, Myg31, and Hkd61.

**Figure 3:**
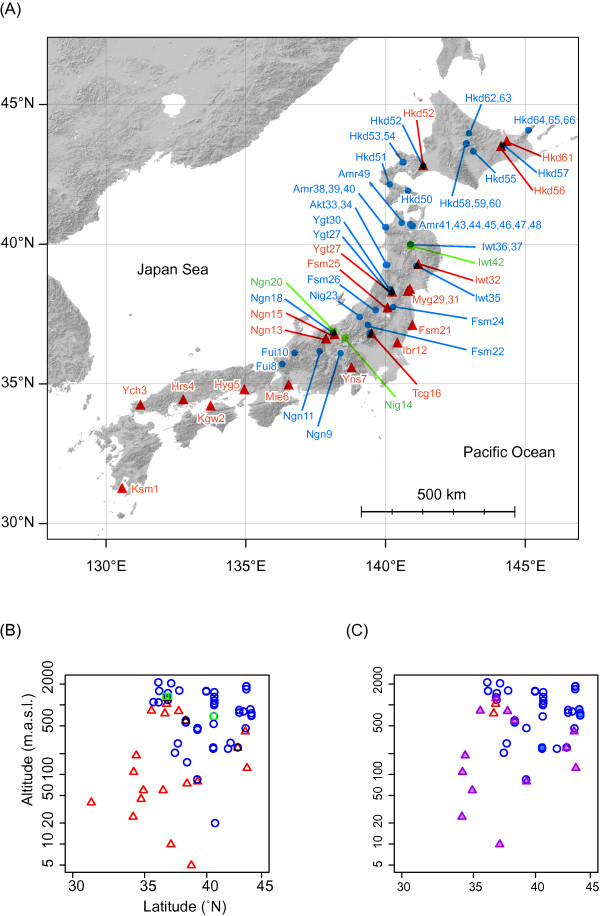
**Geographical locations (A) and distribution patterns (B) of the collected specimens**. (A, B) Red-outlined triangles represent populations possessing *D. galeata *mtDNA. Blue-outlined circles represent populations possessing *D. dentifera *mtDNA. Green-outlined squares represent populations of the new species lineages [see Figure 2]. (C) Purple-filled triangles represent populations possessing haplotypes of *D. galeata *mtDNA and alleles of the *D. dentifera *ITS-1 clade [see Figure 6]. Red-outlined triangles represent populations consisting of individuals possessing haplotypes of *D. galeata *mtDNA and alleles of the *D. galeata *ITS-1 clade. Blue-filled circles represent populations possessing haplotypes of *D. dentifera *mtDNA and alleles of the ITS-1 lineage G3. Blue-outlined circles represent populations consisting of individuals possessing haplotypes of *D. dentifera *mtDNA and alleles of the *D. dentifera *ITS-1 clade.

The mitochondrial 12SrRNA phylogeny revealed that Japanese populations consisted of three major clades: *D. galeata*, *D. dentifera*, and new species lineages (Figure 2). The 12SrRNA alignment (432 bp) was composed of 74 unique haplotypes from 66 Japanese populations with 22 sequences deposited in GenBank [see Additional file 1]. Seven indels were observed at the sites 52, 56, 242, 332, 407, 408, and 413. The new species lineages were found in three populations: Nig14, Ngn20, and Iwt42 (Figure 3).

**Figure 4:**
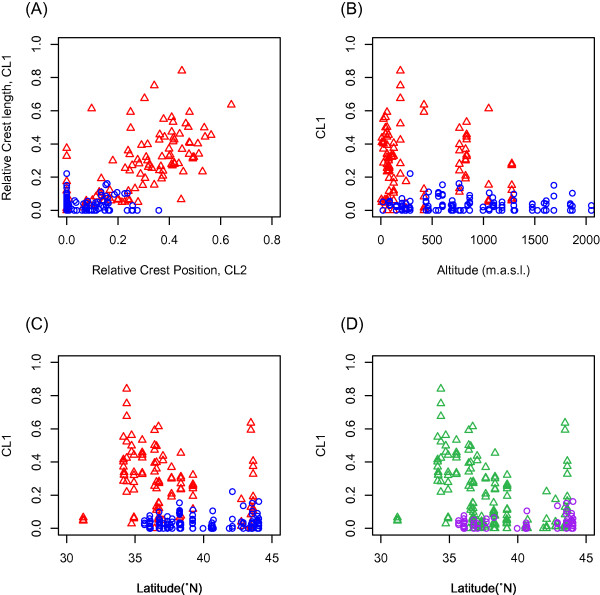
**Relationship between relative length (CL1) and relative position (CL2) of the crest (A), and the relationship between CL1 and latitude (B) and altitude (C, D)**. (A, B, C) Red triangles represent specimens with *D. galeata *mtDNA. Blue circles represent specimens with *D. dentifera *mtDNA. (D) Green triangles and purple circles represent specimens occurred in the presence and absence of fish predators, respectively.

**Figure 5:**
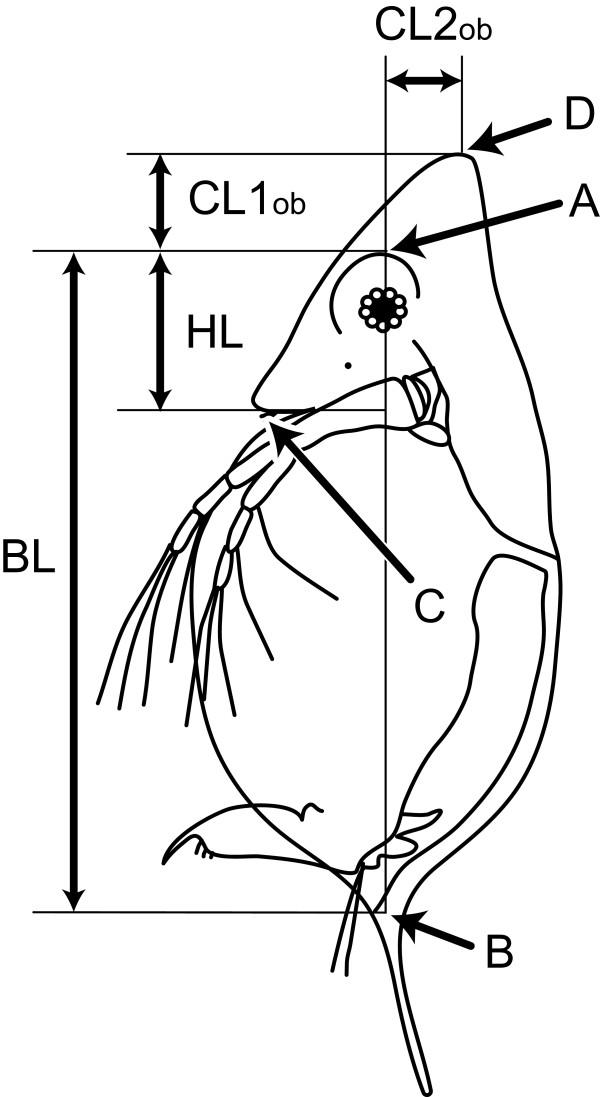
**Measured features of *Daphnia *morphology**. A is the most anterior point of circular compartment around the compound eye. B is the point of tail spine attachment to the carapace. C is the tip of the rostrum. D is the tip of the head crest. Body length (BL) is the length between A and B. Head length (HL) is the length between A and the intersection of line AB and the perpendicular line extending from C to line AB. Crest length (CL1_ob_) is the length between A and the intersection of line AB and the perpendicular line from D to line AB. Crest position (CL2 _ob_) is the length between D and the intersection of line AB and the perpendicular line extending from D to line AB. Relative crest length and position (CL1 and CL2) were computed using the following equations to eliminate the effect of body length: CL1 = ln(CL1 _ob_/HL + 1), CL2 = ln(CL2 _ob_/HL + 1)

Differences in head morphology were observed between specimens with *D. galeata *mtDNA and those with *D. dentifera *mtDNA. While head morphology of specimens with *D. galeata *mtDNA was various, that of specimens with *D. dentifera *mtDNA was characterized as smaller and more ventrally located crest (Figure 4A). Linear discriminant analysis was conducted using three morphological variables: relative length of crest (CL1), relative position of crest (CL2), and body size (BL) (Figure 5). Because misclassification probability was lower when using only CL1 and CL2, BL was not used in this analysis. Scores of linear discriminators (LD1) of specimens with *D. dentifera *mtDNA classified these specimens without error, whereas 19.5% of specimens with *D. galeata *mtDNA were misallocated as those with *D. dentifera *mtDNA (f_LD1 _= 3.636 × CL1 + 6.335 × CL2 - 1.619) [see Additional file 2]. These misallocated specimens with *D. galeata *mtDNA were found, irrespective of latitude and altitude.

We identified a significant positive correlation between CL1 and CL2 of both groups of specimens (specimens with *D. galeata *mtDNA: r = 0.4244, P < 0.0064; specimens with *D. dentifera *mtDNA: r = 0.6759, P < 0.0001; Figure 4A), and a significant negative correlation between BL and CL2 of specimens with *D. galeata *mtDNA (r = -0.571, P < 0.0002) [see Additional file 3].

Differences in geographical distribution were also observed between the two groups of specimens (Figure 3). Specimens with *D. galeata *mtDNA were distributed at lower latitudes and altitudes, whereas those with *D. dentifera *mtDNA were predominately distributed at higher latitudes and altitudes (Figure 3B). Fish were present at 19 collecting sites where specimens with *D. galeata *mtDNA occurred, and 18 sites where specimens with *D. dentifera *mtDNA occurred, whereas fish were absent in only one site (Myg31) where specimens with *D. galeata *mtDNA occurred and 27 sites where specimens with *D. dentifera *mtDNA occurred. Groups with both mtDNA types coexisted in two lakes: Hkd52 and Ygt27. The results of model selection using GLM showed that the presence of specimens with *D. galeata *mtDNA at the collecting site was best explained by the 3 parameters of latitude (L), altitude (A), and fish presence (F) (best model, L + A + F, AIC = 54.33; second model, L + F, AIC = 55.60; third model, L + A, AIC = 56.922), and that the presence of specimens with *D. dentifera *mtDNA was also best explained by latitude (L), altitude (A), and fish absence (F) (best model, L + A + F, AIC = 50.98; second model, L + A, AIC = 51.021; third model, L + F, AIC = 52.671). In the cases of both mtDNA types, if we used only one variable (i.e., any of L, A or F), ΔAIC (difference from the best model) values were larger than 10. Thus, it can be reasonably concluded that latitude, altitude, and the presence or absence of fishes affects the distribution of *D. galeata *and *D. dentifera *mtDNA types.

### Effects of environmental factors on species morphology and distribution

MANOVA tests were performed with identification of mtDNA type (*D. galeata *or *D. dentifera *mtDNA) based on 12SrRNA phylogeny and morphological traits of BL, CL1, and CL2 as the dependent variables, and latitude, altitude, and the presence or absence of fish as the independent variables. The results indicated that the latitude and the presence or absence of fish affected morphology (i.e., BL, CL1, and CL2) and mtDNA types (fish: Wilks' λ = 0.592, F_4,74 _= 12.72, P = 6.30 × 10^-8^; latitude: Wilks' λ = 0.857, F_4,74 _= 3.09, P = 0.021; altitude: Wilks' λ = 0.990, F_4,74 _= 0.178, P = 0.95). Among the four dependent variables, CL1, CL2 and mtDNA types were affected by fish and latitude, and BL was affected only by fish (effect of fish on mtDNA types: F_1,77 _= 51.83, P = 3.43 × 10^-10^; effect of latitude on mtDNA types: F_1,77 _= 8.34, P = 0.005; effect of fish on CL1: F_1,77 _= 25.084, P = 3.40 × 10^-6^; effect of latitude on CL1: F_1,78 _= 4.978, P = 0.028; effect of fish on CL2: F_1,77 _= 29.918, P = 5.40 × 10^-7^; effect of latitude on CL2: F_1,78 _= 11.990, P < 0.0009; effect of fish on BL: F_1,77 _= 4.263, P = 0.043)(Figure 4 B, C and 4D).

### Nuclear ITS-1 phylogeny

**Figure 6:**
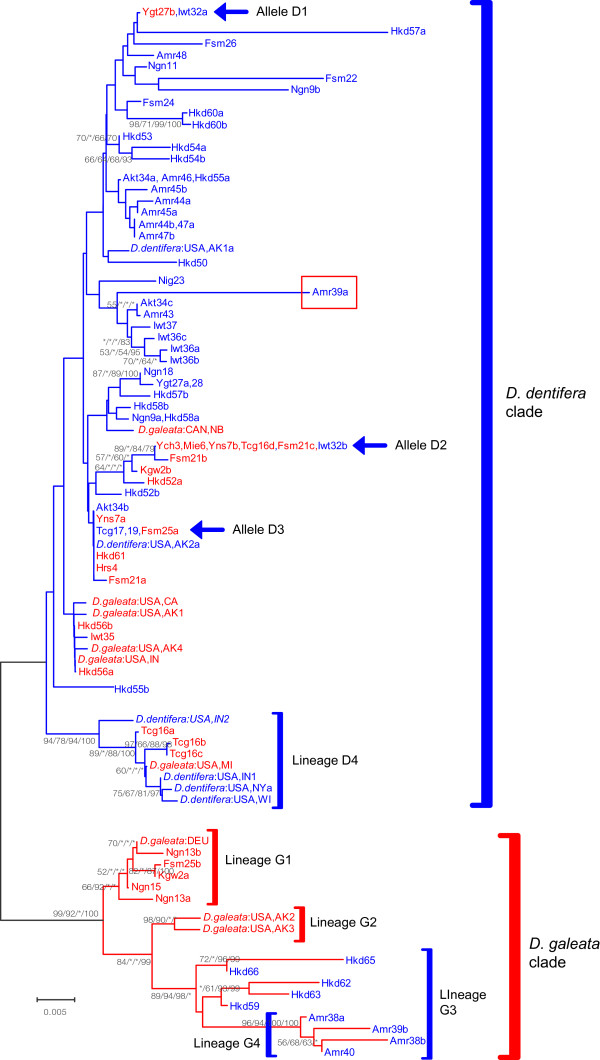
**Neighbor-joining (NJ) phylogram of nuclear ITS-1**. The four numbers on each branch indicate greater than 50% bootstrap support values for the branch as determined by NJ, maximum parsimony, and maximum likelihood methods, and a Bayesian clade credibility value of greater than 70%. Asterisks indicate values with less than 50% bootstrap support values, less than 70% Bayesian support values, or no support. We used red branch lines for the clades of *D. galeata*, blue branch lines for the clades of *D. dentifera*, red characters for the specimens with *D. galeata *mtDNA, and blue characters for the specimens with *D. dentifera *mtDNA. Amr39a in the *D. dentifera *clade was a recombinant sequence between lineage D4 and the *D. dentifera *clade.

Nuclear ITS-1 phylogeny revealed that the Japanese populations of *D. galeata *and *D. dentifera *consisted of two clades: the *D. galeata *clade and the *D. dentifera *clade (Figure 6). This phylogeny is consistent with the nuclear phylogeny of ITS-1 and ITS-2 of Taylor et al. [23]. Since ITS-1 and ITS-2 have a close physical linkage, both phylogenies are expected to reflect the same evolutionary histories. The ITS-1 alignment (675 bp) consisted of 20 unique sequences from 15 populations possessing *D. galeata *mtDNA, 46 unique sequences from 37 populations possessing *D. dentifera *mtDNA, and 15 sequences of *D. galeata *and *D. dentifera *deposited in GenBank [see Additional file 1]. A sequence of an individual obtained from Hkd54 was found to have a 60 bp repeat fragment from position 375 to 435. We removed this repeated fragment for the alignment. An obvious difference between the clades of *D. galeata *and *D. dentifera *was found in a segment between positions 195 and 268.

Recombination test supported the breakpoint at position 192. We compared a phylogeny based on positions 1 to 192 with one based on positions 193 to 675. A sequence of an individual obtained from Amr39 was found in lineage G4 (*D. galeata *clade, Figure 6) based on positions 1 to 192, but matched the *D. dentifera *clade in the phylogeny based on positions 193 to 675, indicating a recombination event between the *D. dentifera *clade and lineage G4. None of the other sequences was found to be recombinant.

The *D. galeata *clade consisted of three lineages: G1, G2, and G3. Lineage G1 consisted of European *D. galeata *and Japanese specimens with *D. galeata *mtDNA. Lineage G2 consisted of Alaskan *D. galeata*. Lineage G3 consisted of specimens with *D. dentifera *mtDNA in Hokkaido Island and Aomori Prefecture, which are the northernmost areas of Japan.

The phylogenetic relationship within the *D. dentifera *clade was not well characterized by bootstrap tests and clade credibility values, with the exception of lineage D4. Alleles D1, D2, and D3 were shared between specimens with *D. galeata *mtDNA and those with *D. dentifera *mtDNA. Linage D4 consisted of North American *D. dentifera *and *D. galeata *and specimens with *D. galeata *mtDNA in Tcg16. All specimens possessing *D. galeata *mtDNA had alleles of the *D. dentifera *clade, except for those of lineage G1 (Figure 3C), while all specimens possessing *D. dentifera *mtDNA had alleles of the *D. dentifera *clade or lineage G3.

### Nuclear ITS-2 RFLP and phylogeny

Five major RFLP patterns (C1, C1+C2, C2, C2+B, and B) were found in the collected specimens of *D. galeata *and *D. dentifera *in Japan. In addition, five minor patterns were found in specimens with *D. galeata *mtDNA in Hyg5, Fsm25, and Myg29, and specimens with *D. dentifera *mtDNA in Amr43 and Ngn9 [see Additional file 4].

We reconstructed ITS-2 phylogeny based on sequence data of the major RFLP patterns from the collected specimens in Japan with sequence data of North American and European specimens deposited in GenBank [see Additional file 5]. The alignment (1111 bp) consisted of 18 sequences from 6 populations possessing *D. galeata *mtDNA, 24 sequences from 9 populations possessing *D. dentifera *mtDNA, and 16 GenBank sequences. Sequences of RFLP pattern B consisted of two clades: one clade included *D. galeata *(i.e., European and North American *D. galeata *and Japanese specimens with *D. galeata *mtDNA) and specimens with *D. dentifera *mtDNA in Ygt27 and Ygt28; the other clade consisted of only Japanese specimens with *D. galeata *mtDNA. Consistent with the assumption of Taylor et al. [23], alleles of RFLP pattern B should be indicative of those derived from *D. galeata *except for the case of Ygt27 and Ygt28. Sequences of RFLP patterns C1 and C2 formed polyphyletic clades. Although sequences of RFLP pattern C1 formed two divergent clades, both of the clades consisted of only specimens with *D. dentifera *mtDNA, indicating that alleles of RFLP pattern C1 should be indicative of those derived from *D. dentifera*, again consistent with the assumption of Taylor et al. (2005).

Allele frequency of RFLP pattern C2 was high in both groups of specimens in Japan, while those of RFLP patterns B and C1 were quite different between the two groups: specimens with *D. galeata *mtDNA (B: 23.4%, C1: 0.8%, C2: 64.5%); specimens with *D. dentifera *mtDNA (B: 13.4%, C1: 9.4%, C2: 73.5%). Specimens with alleles of RFLP patterns C1 and C2 were broadly distributed, irrespective of the latitudinal and altitudinal gradients in Japan.

## Discussion

Phylogenetic differences in mitochondrial 12SrRNA sequences corresponded, for the most part, with the morphological differences in head shape. Specimens with *D. dentifera *mtDNA exhibited less morphological variation, and had either no crest or a smaller more ventrally located crest, suggesting that individuals with *D. dentifera *mtDNA would retain the typical head morphology of *D. dentifera*. On the other hand, specimens with *D. galeata *mtDNA exhibited considerably more variation. Whereas most specimens with *D. galeata *mtDNA had longer and more dorsally located crests, some were indistinguishable from *D. dentifera *due to a less developed crest. The differences in mtDNA also corresponded to distributional differences; specimens with *D. galeata *mtDNA were mostly distributed at lower latitudes and altitudes, while specimens with *D. dentifera *mtDNA were mostly distributed at higher latitudes and altitudes (Figure 3B). The mitochondrial dichotomy between the two *Daphnia *species corresponded well with morphological and distributional differences in Japan.

Morphological traits and geographical distribution patterns were related to the presence or absence of fish, and to latitude. Specimens with *D. dentifera *mtDNA, or specimens with no crest or with smaller crests, were distributed in lakes and ponds without fish predators, or located at higher latitudes. In contrast, specimens with *D. galeata *mtDNA, or specimens with longer and more dorsally located crests, were distributed in lakes and ponds containing fish predators, or located at lower latitudes. Although individuals with larger body size tended to be distributed in lakes and ponds without fish predators, the effect of the presence of fish on head shape was larger than the effect on body size. Habitats located at higher altitudes tend to have fewer fish due to the limited dispersal ability of fish species. They also tend to be lower in temperature, thus regulating the feeding types and activities of predators. Because the differences in defensive strategies between *D. dentifera *and *D. galeata *can promote morphological differentiation in head shape, body size, and ecological differentiation in different habitat types [22], the differences in predation pressures may relate to the parapatric distribution of *D. galeata *and *D. dentifera *into latitudinal and altitudinal gradients in Japan. Since the presence of fish can be an important factor in determining the distributions of the two species, the introduction of fish might diminish the occurrence and diversity of *D. dentifera *in lakes and ponds, particularly those located at higher latitudes. Global warming would also be predicted to reduce the distribution ranges of organisms inhabiting lakes and ponds at higher altitudes, such as *D. dentifera *in Japan.

The discordance between mtDNA and nuclear ITS-1 phylogenies is indicative of interspecific introgression and hybridization between the two species. Specimens with *D. galeata *mtDNA in Tcg16 were found unexpectedly in lineage D4 of the *D. dentifera *ITS-1 clade, which consisted of the alleles of North American *D. dentifera *and *D. galeata*, except for the alleles of Tcg16 (Figure 6), indicating that the alleles of lineage D4 may have recently been introduced from North American populations into Tcg16. Likewise, Ishida & Taylor [33] argued that a mitochondrial haplotype of *D. galeata *in Tcg16 may have been introduced from North America along with the introduction of certain species of fish, such as *Oncorhynchus mykiss *and *Salvelinus namaycush*, which were introduced into Tcg16 in 1877 for commercial and game fishing. Lineage G3 of the *D. galeata *ITS-1 clade consisted of specimens with *D. dentifera *mtDNA in the northernmost areas of Japan, and was more diverse than lineages G1 and G2, indicating incomplete lineage sorting or introgression of common ancestral alleles of lineage G3 from *D. galeata *into *D. dentifera *in the distant past. Since all analyzed specimens with *D. dentifera *mtDNA had ITS-1 alleles of the *D. dentifera *clade or lineage G3, we had no clear evidence for introgression from *D. galeata *into *D. dentifera *in Japan. This is consistent with the smaller amount of variation in head morphology of specimens with *D. dentifera *mtDNA. On the other hand, a discordance between mitochondrial and nuclear phylogeny was frequently observed for specimens with *D. galeata *mtDNA (Figure 3C and 6). The same pattern was observed between these two species in North America [16,23,33].

Nuclear ITS-2 phylogeny also suggested interspecific introgression between the two species. A clade of the alleles of RFLP pattern B (indicative of *D. galeata*) consisted of *D. galeata *(i.e., European and North American *D. galeata *and Japanese specimens with *D. galeata *mtDNA) and specimens with *D. dentifera *mtDNA in two neighbouring ponds of Ygt27 and Ygt28, which are c. 600 meters distant. Although the population of Ygt28 possessed only *D. dentifera *mtDNA, the population of Ygt27 possessed both mtDNA types of *D. galeata *and *D. dentifera*, suggesting that hybridization or introgression between the species should occur within the specific region around Ygt27 and Ygt28.

## Conclusion

We tested a hypothesis that the long history of coexistence between *D. galeata *and *D. dentifera *in Japan has led to extensive genetic admixture in both nuclear and mitochondrial DNA, despite the fact that differences in the morphological traits related to environmental factors, such as predation pressure, have been maintained. In the present study, the correspondence observed among morphology, distribution, and mitochondrial and nuclear DNA types for the specimens with *D. dentifera *mtDNA suggests that the species distinction has been maintained for *D. dentifera*. However, discordance between mtDNA and nuclear ITS-1 types was observed for most specimens with *D. galeata *mtDNA, consistent with the pattern found in North America. This suggests nuclear introgression from *D. dentifera *into *D. galeata *without mitochondrial introgression. The results indicate that variations in environmental factors, such as predation pressure and temperature, have maintained the separation of distributional ranges and morphological differences, such as head shape, between the two species. However, we cannot rule out other factors, and further studies are needed to verify the hypothesis that selective pressure has maintained the morphological differences between the two species. We were unable to estimate the frequency of the introgression, and we were thus unable to conclude that the long history of coexistence between *D. galeata *and *D. dentifera *in Japan has caused more frequent introgression between the two species than the frequency observed in the same species in North America. However, even though climatic and geographical conditions have differed greatly between North America and Japan, particularly during the ice ages, differences in the morphological traits related to environmental factors, such as predation pressure, have been maintained in both regions. The pattern of introgression was also consistent between the two regions. Since the present study indicated that the presence of fish can be an important factor in determining the distributions of the two species, fish introduction can also diminish the occurrence and diversity of *D. dentifera *in lakes and ponds, particularly those located at higher latitudes. Global warming would also be likely to reduce the distribution ranges of organisms inhabiting lakes and ponds at high altitude, such as *D. dentifera *in Japan,

## Methods

### Sample collection

Specimens of *D. galeata *and *D. dentifera *were collected from 66 populations (lakes and ponds) in Japan [see Additional file 1]. Distribution of planktivorous fishes in given ponds/lakes was examined by interviews for local peoples, anglers and fishermen, and records in research and historical documents of local towns and villages. The planktivorous fishes we examined were cyprinids such as minnows and crucian carps, salmonid and smelts which are known to prey preferentially *Daphnia *species among zooplankton (e.g., [40]). All samples were preserved in absolute ethanol or methanol at 4°C. Mitochondrial phylogeny clearly distinguished clades of *D. galeata *and *D. dentifera *[8,14,21,23,33]. We analyzed mitochondrial 12S sequences of two to four collected specimens from each population, and confirmed that most of populations consisted of either specimens with *D. galeata *mtDNA or those with *D. dentifera *mtDNA. Two populations consisted of both specimens with *D. galeata *mtDNA and with *D. dentifera *mtDNA. We sequenced 9 to 10 specimens from each of the two populations, and identified mtDNA types of each specimen. Other three populations consisted of specimens of new mitochondrial lineages, which are different from any known lineages of the subgenus *Hyalodaphnia*. We excluded the populations of the new species lineages from the analyses. Then, we performed the analyses of nuclear phylogeny, RFLP, and morphological traits. First, we compared morphologies between specimens with *D. galeata *mtDNA and those with *D. dentifera *mtDNA (see the later section of morphological measurements) to examine to what extent the mtDNA types can be distinguished by morphology. Then, linear discriminant analysis was also performed to quantify whether and to what extent morphologies determine the mtDNA types, with the aid of the statistical software R 2.8.1 (R Development Core Team).

We applied model selection using generalized linear regression (GLM) to examine which variables (latitude, altitude, presence/absence of fishes, and their interaction terms) affect the presence or absence of *D. dentifera *or *D. galeata *in the collecting site. Since the dependent variable was binomial (i.e., presence or absence), we used GLM with binominal distribution and logit link. We explored the set of predictors of the independent variables with a stepwise AIC procedure; independent variables were removed and added to the models to determine the set of predictors that yielded the lowest AIC by using the software R.

### DNA extraction, PCR, and sequencing

Total genomic DNA was extracted using a chelating resin, Chelex 100 (Bio-Rad). Samples were incubated in 30 μL of 6% Chelex 100 at 60°C for 3 h, mixed for a short period, incubated at 98°C for 10 min, centrifuged at 14,000 rpm at 4°C for 1 min, stored at RT overnight, and diluted 10 times with TE buffer. We amplified a fragment of mitochondrial 12SrRNA using the primers 5'- ATG CAC TTT CCA GTA CAT CTA C -3' and 5'- AAA TCG TGC CAG CCG TCG C -3' [14], and a fragment of nuclear ITS-1 using the 18SD primer 5' - CAC ACC GCC CGT CGC TAC TAC CGA TTG -3' and the 5.8BR primer 5'- TAG GAT TAG CGC ACT TTG CTG C -3' [23]. Each 50 μL of polymerase chain reaction (PCR) mixture consisted of 5 μL of extracted DNA, 0.5 μM of each primer, 0.2 mM of each dNTP, 10 mM of Tris HCl (pH 8.3), 5 mM of MgCl_2_, 50 mM of KCl, and 0.25 units of rTaq (Takara). The PCR temperature profile used for both the 12SrRNA and ITS-1 amplification reactions was as follows: 1 cycle of 94°C for 1 min, 10 cycles of 94°C for 1 min, 53°C for 1 min, and 72°C for 2 min, and 30 cycles of 92°C for 1 min, 53°C for 2 min, and 72°C for 1 min. After cycle-sequencing using a Big Dye Terminator Cycle Sequencing Kit (Applied Biosystems), sequences were obtained using an ABI PRISM 3130-Avant Genetic Analyzer (Applied Biosystems), aligned with Clustal X [41], and manually adjusted with Se-Al 2.0 [42].

### ITS-2 RFLP

We discriminated nuclear ITS-2 sequences of specimens by using the RFLP method of Taylor et al. (2005). A fragment of ITS-2 was amplified using the 5.8BF primer 5'- ACC CTG AAC GGT GGA TCA CTA GGC TC -3' and the 28SD2BR primer 5'- TTA GAA GGA GTT TAC CTC CCG CTT AGG -3'. Each 10 μL of the PCR mixture consisted of 5 μL of extracted DNA, 0.5 μM of each primer, 0.2 mM of each dNTP, 10 mM of Tris HCl (pH 8.3), 5 mM of MgCl_2_, 50 mM of KCl, and 0.25 units of rTaq (Takara). The PCR temperature profile for the ITS-2 amplification reaction was: 40 cycles of 94°C for 1 min, 58°C for 1 min, and 72°C for 2 min, and final extension at 72°C for 20 min. Next, 2.5 μL of PCR products were digested with *Rsa*I (New England Biolabs) at 37°C for 3-6 h. Digested products were then electrophoresed on a 2.5% agarose gel in the presence of ethidium bromide, and classified into five major RFLP patterns (C1, C2, B, C1+C2, C2+B) and other minor patterns, according to Taylor et al. (2005). The C1+C2 and C2+B patterns indicate the heterozygote of the C1 and C2 alleles and the heterozygote of the C2 and B alleles, respectively. We analyzed 138 individuals from 43 populations possessing *D. dentifera *mtDNA, and 62 individuals from 20 populations possessing *D. galeata *mtDNA [see Additional file 1]. Two or three individuals of each major RFLP pattern, representing a total of 15 individuals from 15 populations, were cloned and sequenced. Cloning was performed using a TOPO TA Cloning Kit (Invitrogen). Then, three cultured colonies were used for sequencing. After cycle-sequencing with a Big Dye Terminator Cycle Sequencing Kit, sequences were obtained using an ABI PRISM 3130-Avant Genetic Analyzer, aligned with Clustal X, and manually adjusted with Se-Al 2.0.

### Reconstruction of phylogeny

We analyzed 205 individuals from 66 populations for mitochondrial 12SrRNA phylogeny using sequences of the subgenus *Hyalodaphnia *deposited in GenBank [see Additional file 1]. After we identified the specimens as either *D. galeata *mtDNA type or *D. dentifera *mtDNA type on the basis of 12SrRNA phylogeny, we directly sequenced and analyzed the nuclear ITS-1 region of 31 individuals from 15 populations possessing *D. galeata *mtDNA type and 60 individuals from 37 populations possessing *D. dentifera *mtDNA type. We also analyzed the cloned sequences of the nuclear ITS-2 region of 15 individuals from 15 populations possessing either *D. galeata *mtDNA or *D. dentifera *mtDNA. We reconstructed nuclear phylogenies of ITS-1 and ITS-2 with the sequences of *D. galeata *and *D. dentifera *deposited in GenBank using the methods of neighbor-joining (NJ), maximum parsimony (MP), maximum likelihood (ML), and Bayesian inference (BI). NJ methods were performed in the program MEGA 5 [43] employing Kimura 2 parameters models (K80) of sequence evolution [44], pairwise gap deletions, and 1000 bootstrap replicates. MP methods were performed in the program MEGA 5 employing a close neighbour interchange as search option (level = 1), complete gap deletions and 1000 bootstrap replicates. ML analyses were performed in the program RAxML [45] with the General Time Reversible (GTR) plus gamma distributed model of sequence evolution and 1000 rapid bootstrap replicates [46]. BI analyses were performed in the program MrBayes 3.1.2 [47] using models of sequence evolution independently selected for 12S, ITS-1, and ITS-2 data sets by hierarchical likelihood ratio tests in MrModeltest 2.3 [48]. Selected models for 12S, ITS-1, ITS-2 were GTR plus inverse gamma distribution, K80 plus inverse gamma distribution, K80 plus gamma distribution, respectively. Four Markov chains were started from randomly chosen trees and run for 6,000,000 generations for 12S, for 17,500,000 generations for ITS-1, and for 2,000,000 generations for ITS-2 with sampling every 100 generations. We confirmed convergence of the Markov chains by examining whether average standard deviation of split frequencies was under 0.1 and whether potential scale reduction factor was close to 1.0. Screening for recombinant sequences in the nuclear ITS-1 and ITS-2 alignment was performed using single breakpoint algorithm [49].

### Morphological measurements

We measured head length (HL), crest height (CL1_Ob_), and crest position (CL2_Ob_) (Figure 5) of 183 individuals from 43 populations of *D. dentifera *mtDNA type and 108 individuals from 19 populations of *D. galeata *mtDNA type [see Additional file 1]. In addition, we measured body size (BL) of 41 individuals from 15 populations of *D. dentifera *mtDNA type and 40 individuals from 12 populations of *D. galeata *mtDNA type, which are a subset of the samples measured for HL, CL1_Ob_, and CL2_Ob_. Each individual was photographed and measured using image J (NIH). The relative height (CL1) and relative position (CL2) of the crest were computed using the following equation to eliminate the effect of head length (HL) on the observed height (CL1_Ob_) and the position (CL2_Ob_) of the crest:

A larger value of CL1 indicates that the crest is more elongated, and a larger value of CL2 means that the crest is located closer to the dorsal margin.

To address the effects of latitude, altitude and existence of fish predators on morphology and mtDNA type, we performed multivariate analysis of variance (MANOVA) by designating the latitude, altitude, and the existence of fish predators (1 or 0) as independent variables and mtDNA types, CL1, CL2 and as dependent variables. Each statistical analysis was performed using the software R.

## Authors' contributions

SI and AT carried out the molecular genetic lab work and the genetic and statistical analyses, participated in the design of the study and drafted the manuscript. NM and JY participated in the lab work and helped to draft the manuscript. WM collected samples for the analyses and helped to draft manuscript. JU collected samples for the analyses, participated in the design of the study and helped to draft the manuscript. MK supervised the design of the study, performed statistical analyses and drafted the manuscript. All authors read and approved the final manuscript.

## Supplementary Material

Additional file 1 **List of *Daphnia *specimens subjected to genetic and morphological analyses**. List A shows information on the Japanese specimens used for genetic and morphological analyses, and list B shows information on the North American and European specimens used for genetic analyses. Click here for file

Additional file 2 **Frequency distribution of individuals of the scores of linear disciminators for mitochondrial *D. dentifera *(above panel) and *D. galeata *(below panel)**. Upper panel shows the frequency distribution of the specimens with *D. dentifera *mtDNA, and lower panel shows that of the specimens with *D. galeata *mtDNA. Click here for file

Additional file 3 **Relationship between body size (BL), relative crest length (CL1), and relative crest position (CL1)**. (A) A plot of the relationship between BL and CL2. (B) A plot of the relationship between BL and CL1. Red triangles and blue circles represent specimens with *D. galeata *mtDNA and those with *D. dentifera *mtDNA, respectively. Click here for file

Additional file 4 **An image of an agarose gel with the five main patterns (C1, C1+C2, C2, C2+B, and B) and the five minor patterns of specimens with *D. galeata *mtDNA in Hyg5, Fsm25, and Myg29, and specimens with *D. dentifera *mtDNA in Amr43 and Ngn9**. The patterns were obtained by digesting the nuclear ITS-2 region with the *Rsa*I restriction enzyme. The last lane is a size ladder with rungs from 300 bp to 1000 bp. Click here for file

Additional file 5 **Neighbor-joining (NJ) phylogram of the nuclear ITS-2**. The four numbers on each branch indicate greater than 50% bootstrap support values for the branch as determined by NJ, maximum parsimony, and maximum likelihood methods, and a Bayesian clade credibility value of greater than 70%. Asterisks indicate values with less than 50% bootstrap support values, less than 70% Bayesian support values, or no support. We distinguished the color of the branch line based on the ITS-1 RFLP patterns (B, C1, and C2). We used a red line for the sequences of pattern B, a blue line for those of pattern C1, a gold line for those of pattern C2, red characters for the specimens with *D. galeata *mtDNA, and blue characters for those with *D. dentifera *mtDNA. Click here for file
