# Supplementary material for: The long-term consequences of hybridization between the two Daphnia species, D. galeata and D. dentifera, in mature habitats
Source: BMC Evol Biol. 2011 Jul 15;11:209. doi: 10.1186/1471-2148-11-209 (PMC3156774; doi:10.1186/1471-2148-11-209)
Supplement: Additional file 1 — List of Daphnia specimens subjected to genetic and morphological analyses. List A shows information on the Japanese specimens used for genetic and morphological analyses, and list B shows information on the North American and European specimens used for genetic analyses. [file 1471-2148-11-209-S1.PDF]

Additional file 1 –List of *Daphnia* specimens subjected to genetic and morphological analyses

List A shows the information of Japanese specimens used for genetic and morphological analyses, and list B shows the information of North American and European specimens used for genetic analyses.

(A)

ID	Location	Latitude	Longitude	Altitude (m.a.s.l.)	Fish	Genetic analyses															Morphological analyses	
						Mitochondrial 12S rRNA			Nuclear ITS-1 region			Nuclear ITS-2 region										
						MtDNA type	No. of analyzed individuals	Haplotypes (A number in parenthesis indicates number of analyzed individuals posessing the sequence in the population) [Characters in bracket indicates DDBJ accession number of the sequence]	No. of analyzed individuals	Sequences of *D. dentifera* type (A number in parenthesis indicates number of analyzed individuals posessing the sequence in the population) [Characters in bracket indicates DDBJ accession number of the sequence]	Sequences of *D. galeata* type. (A number in parenthesis indicates number of analyzed individuals posessing the sequence in the population, and characters in bracket indicates DDBJ accession number of the sequence.)	No. of individuals used for RFLP analysis	No. of individuals of C1 pattern	No. of individuals of C1+C2 pattern	No. of individuals of C2 pattern	No. of individuals of C2+B pattern	No. of individuals of B pattern	No. of individuals of minor patterns	RFLP pattern of the individuals used for cloning and sequencing	Sequences (Characters in bracket indicate DDBJ accession number of the sequence.)	No. of individuals measured for crest length	No. of individuals measured for crest length and body size
Ksm1	Lake Kagami-ike, Kagoshima	31.2106˚N	130.5556˚E	40	present	*D. galeata*	3	Ksm1(3)[AB642029]	0			3			2	1				gKsm1a [AB642085], gKsm1b [AB642086], gKsm1c [AB642087]	3	0
Kgw2	A pond in Sanuki-Toyonaka, Kagawa	34.1458˚N	133.7150˚E	25	present	*D. galeata*	3	Kgw2(3)[AB642028]	3	Kgw2b(1)[AB641964]	Kgw2a(2)[AB641963]	3			1		2		B	gKgw2a [AB642082], gKgw2b [AB642083], gKgw2c [AB642084]	5	3
Ych3	A pond in Raihukudai, Yamaguchi	34.1869˚N	131.1997˚E	110	present	*D. galeata*	3	Ych3a(2)[AB642042], Ych3b(1)[AB642043]	2	Ych3(2)[AB641971]		2			2						3	0
Hrs4	Oginoike Pond, Hiroshima	34.3819˚N	132.7369˚E	190	present	*D. galeata*	3	HaplotypeA(3)[AB641994]	1	Hrs4(1)[AB641961]		3			2		1				7	4
Hyg5	Mizogasawa Pond, Hyogo	34.7392˚N	134.9264˚E	45	present	*D. galeata*	3	HaplotypeA(3)[AB641994]	0			4			2			2			4	0
Mie6	Suzuka-Osawa Pond, Mie	34.9108˚N	136.5189˚E	60	present	*D. galeata*	3	HaplotypeA(2)[AB641994], Mie6(1)[AB642028]	1	Mie6(1)[AB641971]		3			3						7	3
Yns7	Lake Kawaguchi, Yamanashi	35.5168˚N	138.7599˚E	835	present	*D. galeata*	3	Yns7a(2)[AB642029], Yns7b(1)[AB642048]	2	Yns7a(1)[AB641974], Yns7b(1)[AB641971]		6			2	4			C2+B	gYns7a [AB642088], gYns7b [AB642089], gYns7c [AB642090]	6	3
Fui8	Lake Yasyagaike, Fukui	35.6725˚N	136.2903˚E	1,100	absent	*D. dentifera*	3	Fui8a(2)[AB641992], Fui8b(1)[AB641993]	0			3			3				C2	dFui8a [AB642059], dFui8b [AB642060]	3	0
Ngn9	Lake Shirakoma, Nagano	36.0513˚N	138.3697˚E	2,115	absent	*D. dentifera*	3	Ngn9_18(3)[AB642034]	2	Ngn9a(1)[AB641949], Ngn9b(1)[AB641950]		7		1	2			4			5	2
Fui10	Lake Karikomi-ike, Fukui	36.0681˚N	136.7275˚E	1,090	absent	*D. dentifera*	3	Fui10(3)[AB641991]	0			3			3						3	0
Ngn11	Lake Ushitame-ike, Nagano	36.1136˚N	137.6144˚E	1,590	absent	*D. dentifera*	3	Ngn11(3)[AB642030]	1	Ngn11(1)[AB641947]		3			3				C2	dNgn11a [AB642061], dNgn11b [AB642062], dNgn11c [AB642063]	3	0
Ibr12	Kouzogawa Reservoir, Ibaragi	36.4183˚N	140.3956˚E	60	present	*D. galeata*	3	HaplotypeA(3)[AB641994]	0			4			4						7	5
Ngn13	Lake Kizaki, Nagano	36.5584˚N	137.8386˚E	765	present	*D. galeata*	3	HaplotypeA(1)[AB641994], Ngn13(2)[AB642029]	2		Ngn13a(1)[AB641965] ,Ngn13b(1)[AB641966]	3				3					7	4
Nig14	Lake Imori-ike, Niigata	36.6314˚N	138.5361˚E	1,335	absent	new species lineages	3	Nig14(3)[AB642035]	0			0									3	0
Ngn15	Daizahoushi Pond, Nagano	36.7056˚N	138.1439˚E	1,050	present	*D. galeata*	3	Ngn15(3)[AB642031]	2		Ngn15(2)[AB641967]	2					2				7	3
Tcg16	Lake Chuzenji, Tochigi	36.7412˚N	139.4743˚E	1,275	present	*D. galeata*	3	Tcg16a(1)[AB642037], Tcg16b(2)[AB642038]	4	Tcg16b(1)[AB641969], Tcg16c(1)[AB641970], Tcg16d(1)[AB641971], Tcg16a(1)[AB641968]		3			3						8	3
Tcg17	Lake Sainoko, Tochigi	36.7429˚N	139.4058˚E	1,295	present	*D. dentifera*	3	Tcg17a(2)[AB642039], Tcg17b(1)[AB642040]	2	Tcg17(2)[AB641952]		3		1	2				C1+C2	dTcg17a [AB642064], dTcg17b [AB642065], dTcg17c [AB642066]	6	0
Ngn18	Lake Kurohime-Huruike, Nagano	36.7889˚N	138.1069˚E	1,180	absent	*D. dentifera*	2	Ngn18(2)[AB642034]	3	Ngn18(3)[AB641948]		3			3						6	3
Tcg19	Lake Yunoko, Tochigi	36.7987˚N	139.4287˚E	1,475	present	*D. dentifera*	2	Tcg19(2)[AB642041]	2	Tcg19(2)[AB641952]		2			2						5	0
Ngn20	Sennin-ike Pond, Nagano	36.8533˚N	138.1097˚E	1,335	absent	new species lineages	3	Ngn20a(2)[AB642032], Ngn20b(1)[AB642033]	0			0									4	0
Fsm21	Kaneko-tei Pond, Fukushima	37.0339˚N	140.9467˚E	10	present	*D. galeata*	3	HaplotypeA(3)[AB641994]	3	Fsm21a(1)[AB641954], Fsm21b(1)[AB641955], Fsm21c(1)[AB641971]		3			3				C2	gFsm21a [AB642073], gFsm21b [AB642074], gFsm21c [AB642075]	3	0
Fsm22	Chumon-ooike Pond, Fukushima	37.0583˚N	139.3469˚E	2,050	absent	*D. dentifera*	3	Fsm22a(2)[AB641986], Fsm22b(1)[AB641987]	1	Fsm22(1)[AB641973]		3	3						C1	dFsm22a [AB642056], dFsm22b [AB642057], dFsm22c [AB642058]	8	2
Nig23	Kagami-ike Pond, Niigata	37.3567˚N	139.0583˚E	205	present	*D. dentifera*	3	Nig23(3)[AB642036]	1	Nig23(1)[AB641951]		3			3						7	3
Fsm24	Nozawa-no-onuma Pon, Fukushima	37.5922˚N	139.6203˚E	280	present	*D. dentifera*	3	Fsm24(3)[AB641988]	1	Fsm24(1)[AB641925]		2			2						2	0
Fsm25	Lake Hibara, Fukushima	37.6564˚N	140.0551˚E	825	present	*D. galeata*	3	Fsm25(2)[AB641989], HaplotypeA(1)[AB641994]	2	Fsm25a(1)[AB641952]	Fsm25b(1)[AB641956]	6				1	1	4			5	0
Fsm26	A pond in Torikodaira moor, Fukushima	37.7050˚N	140.2481˚E	1,610	absent	*D. dentifera*	3	Fsm26(3)[AB641990]	2	Fsm26(2)[AB641926]		2				2					4	0
Ygt27	Lake Shirataka-aranuma, Yamagata	38.2353˚N	140.2114˚E	600	present	*D. dentifera*	9	Ygt27a(7)[AB642044], Ygt27b(1)[AB642045], Ygt27c(1)[AB642046]	1	Ygt27a(1)[AB641953]		7			1	5	1		B	dYgt27a [AB642067], dYgt27b [AB642068], dYgt27c [AB642069]	6	0
Ygt27	Lake Shirataka-aranuma, Yamagata	38.2353˚N	140.2114˚E	600	present	*D. galeata*	1	HaplotypeA(1)[AB641994]	1	Ygt27b(1)[AB641972]		1					1				1	0
Ygt28	Lake Shirataka-onuma, Yamagata	38.2450˚N	140.2047˚E	560	present	*D. dentifera*	3	Ygt28(3)[AB642045]	1	Ygt28(1)[AB641953]		3			2		1		B	dYgt28a [AB642070], dYgt28b [AB642071], dYgt28c [AB642072]	9	3
Myg29	Bouzu-tei Pond, Miyagi	38.3389˚N	140.8433˚E	75	present	*D. galeata*	2	HaplotypeA(2)[AB641994]	0			3			2			1			7	3
Ygt30	Umagami Pond, Yamagata	38.3431˚N	140.1483˚E	150	present	*D. dentifera*	3	Ygt30a(2)[AB642044], Ygt30b(1)[AB642047]	0			3			3						7	3
Myg31	Miyagi-nagamuma Pond, Miyagi	38.2573˚N	140.8569˚E	5	absent	*D. galeata*	3	HaplotypeA(3)[AB641994]	0			3		1	2						1	0
Iwt32	Kouyano-tei Pond, Iwate	39.1919˚N	141.0717˚E	85	present	*D. dentifera*	3	Iwt32(3)[AB642021]	2	Iwt32b(1)[AB641971], Iwt32a(1)[AB641972]		3				1	2				6	0
Akt33	Nogiwa-tameike Pond, Akita	39.2011˚N	140.0411˚E	440	present	*D. dentifera*	3	Akt33(3)[AB641975]	0			3				3			C2+B	dAkt33 [AB642049]	4	0
Akt34	A pond in Nikaho-Kogen, Akita	39.2169˚N	140.0025˚E	465	present	*D. dentifera*	3	Akt34a(2)[AB641975], Akt34b(1)[AB642046]	3	Akt34a(1)[AB641909], Akt34c(1)[AB641911], Akt34b(1)[AB641910]		3				2	1				4	0
Iwt35	Umakai Pond, Iwate	39.2222˚N	141.1536˚E	80	present	*D. galeata*	3	Iwt35a(1)[AB642022], Iwt35b(2)[AB642038]	1	Iwt35(1)[AB641962]		3			2		1				7	3
Iwt36	Gama-numa Pond, Iwate	39.9554˚N	140.8616˚E	1,590	absent	*D. dentifera*	3	Iwt36a(1)[AB642023], Iwt36b(2)[AB642024]	3	Iwt36a(1)[AB641943], Iwt36b(1)[AB641944], Iwt36c(1)[AB641945]		3			3						1	0
Iwt37	Hachiman-numa Pond, Iwate	39.9565˚N	140.8693˚E	1,560	absent	*D. dentifera*	3	Iwt37a(2)[AB642025], Iwt37b(1)[AB642024]	1	Iwt37(1)[AB641946]		3			3						0	0
Amr38	Nagaike Pond, Aomori	40.5575˚N	139.9789˚E	245	present	*D. dentifera*	3	Amr38a(2)[AB641976], Amr38b(1)[AB641977]	2		Amr38a(1)[AB641912] ,Amr38b(1)[AB641913]	3	3							dAmr38a [AB642050], dAmr38b [AB642051], dAmr38c [AB642052]	6	3
Amr39	Mitchishiba-no-ike Pond, Aomori	40.5583˚N	139.9750˚E	245	absent	*D. dentifera*	3	Amr39a(1)[AB641976], Amr39b(2)[AB641978]	2		Amr39a(1)[AB641914] ,Amr39b(1)[AB641915]	3	3						C1		1	0
Amr40	Ketoba-no-ike Pond, Aomori	40.5628˚N	139.9859˚E	235	present	*D. dentifera*	4	Amr40a(3)[AB641979], Amr40b(1)[AB641982]	1		Amr40(1)[AB641916]	3	3						C1	dAmr40a [AB642053], dAmr40b [AB642054], dAmr40c [AB642055]	0	0
Amr41	Hakkoda-nagamuma Pond, Aomori	40.5958˚N	140.9522˚E	535	absent	*D. dentifera*	3	Amr41(3)[AB641980]	0			3			3						5	3
Iwt42	Hourai-numa Pond, Iwate	40.6096˚N	140.9390˚E	685	absent	new species lineages	2	Iwt42a(1)[AB642026], Iwt42b(1)[AB642027]	0			0									0	0
Amr43	Aka-numa Pond, Aomori	40.6096˚N	140.9390˚E	685	absent	*D. dentifera*	3	Amr43(3)[AB641981]	1	Amr43(1)[AB641917]		8			4	3		1			3	0
Amr44	Suiren-numa Pond, Aomori	40.6327˚N	140.8931˚E	995	absent	*D. dentifera*	3	Amr44(3)[AB641982]	2	Amr44b(1)[AB641919], Amr44a(1)[AB641918]		3			3						2	0
Amr45	Senninn-numa Pond, Aomori	40.6513˚N	140.8843˚E	1,305	absent	*D. dentifera*	3	Amr45(3)[AB641982]	2	Amr45a(1)[AB641920], Amr45b(1)[AB641921]		3			3						3	0
Amr46	Kagami-numa Pond, Aomori	40.6575˚N	140.8842˚E	1,525	absent	*D. dentifera*	3	Amr46(3)[AB641983]	1	Amr46(1)[AB641922]		3			3						3	0
Amr47	A pool in Kamikenashi moor, Aomori	40.6648˚N	140.8666˚E	1,150	absent	*D. dentifera*	3	Amr47(3)[AB641982]	3	Amr47a(1)[AB641919], Amr47b(2)[AB641923]		3			3						2	1
Amr48	A pool in Shimokenashi moor, Aomori	40.6658˚N	140.8573˚E	1,055	absent	*D. dentifera*	3	Amr48(3)[AB641984]	1	Amr48(1)[AB641924]		3			2	1					3	0
Amr49	Pond Kumasawa-tameike, Aomori	40.7186˚N	140.5494˚E	20	present	*D. dentifera*	3	Amr49(3)[AB641985]	0			3			2	1					2	0
Hkd50	Shin-nakano reservoir, Hokkaido	41.8719˚N	140.7866˚E	235	present	*D. dentifera*	3	Hkd50a(1)[AB641995], Hkd50b(1)[AB641996], Hkd50c(1)[AB641997]	1	Hkd50(1)[AB641927]		0									3	0
Hkd51	Ainumanai reservoir, Hokkaido	42.1008˚N	140.1334˚E	285	present	*D. dentifera*	3	Hkd51a(2)[AB641998], Hkd51b(1)[AB641999]	0			3			3						5	3
Hkd52	Lake Shikotsu, Hokkaido	42.7550˚N	141.3226˚E	245	present	*D. dentifera*	4	Hkd52a(2)[AB641998], Hkd52b(1)[AB642000], Hkd52c(1)[AB642001]	1	Hkd52b(1)[AB641928]		4			4				B		2	0
Hkd52	Lake Shikotsu, Hokkaido	42.7550˚N	141.3226˚E	245	present	*D. galeata*	5	Hkd52d(1)[AB642002], Hkd52e(4)[AB642048]	4	Hkd52a(4)[AB641957]		2					2			gHkd52a [AB642076], gHkd52b [AB642077], gHkd52c [AB642078]	3	0
Hkd53	Niseko-naganuma Pond, Hokkaido	42.8952˚N	140.5894˚E	775	absent	*D. dentifera*	3	Hkd53a(1)[AB642003], Hkd53b(2)[AB642004]	1	Hkd53(1)[AB641929]		1			1						6	3
Hkd54	Niseko-onuma Pond, Hokkaido	42.8961˚N	140.6209˚E	845	absent	*D. dentifera*	3	Hkd54(3)[AB642005]	2	Hkd54a(1)[AB641930], Hkd54b(1)[AB641931]		0									2	0
Hkd55	Lake Shikaribetsu, Hokkaido	43.2803˚N	143.1234˚E	805	present	*D. dentifera*	3	Hkd55a(1)[AB642006], Hkd55b(1)[AB642007], Hkd55c(1)[AB642008]	3	Hkd55b(2)[AB641932], Hkd55a(1)[AB641922], Hkd55b(2)[AB641932]		1			1						2	0
Hkd56	Lake Akan, Hokkaido	43.4522˚N	144.1014˚E	420	present	*D. galeata*	3	Hkd56a(1)[AB642009], Hkd56b(2)[AB642038]	2	Hkd56a(1)[AB641958], Hkd56b(1)[AB641959]		3			3				C2	gHkd56a [AB642079], gHkd56b [AB642080], gHkd56c [AB642081]	7	3
Hkd57	Lake Akan-Panketoh, Hokkaido	43.4831˚N	144.1808˚E	461	present	*D. dentifera*	4	Hkd57a(2)[AB642010], Hkd57b(1)[AB642011], Hkd57c(1)[AB642012]	2	Hkd57b(1)[AB641934], Hkd57a(1)[AB641933]		3					3				6	0
Hkd58	Ama-numa Pond, Hokkaido	43.5475˚N	142.8527˚E	1,845	absent	*D. dentifera*	3	Hkd58a(2)[AB642013], Hkd58b(1)[AB642014]	2	Hkd58a(1)[AB641949], Hkd58b(1)[AB641935]		4			4						3	0
Hkd59	Hisa-numa Pond, Hokkaido	43.5498˚N	142.8776˚E	1,685	absent	*D. dentifera*	3	Hkd59(3)[AB642015]	1		Hkd59(1)[AB641936]	4			4						5	0
Hkd60	A pool in Keundaira moor, Hokkaido	43.5644˚N	142.8720˚E	1,865	absent	*D. dentifera*	2	Hkd60(2)[AB642016]	2	Hkd60a(1)[AB641937], Hkd60b(1)[AB641938]		3			2	1					5	3
Hkd61	Lake Kussaro, Hokkaido	43.6161˚N	144.3208˚E	125	present	*D. galeata*	3	HaplotypeA(1)[AB641994], Hkd61(2)[AB642022]	1	Hkd61(1)[AB641960]		2			2						10	3
Hkd62	A pool in Ukishima South, Hokkaido	43.9294˚N	142.9569˚E	865	absent	*D. dentifera*	2	Hkd62a(1)[AB642017], Hkd62b(1)[AB642018]	1		Hkd62(1)[AB641939]	3			3						5	3
Hkd63	A pool in Ukishima East, Hokkaido	43.9319˚N	142.9644˚E	865	absent	*D. dentifera*	3	Hkd63(3)[AB642017]	1		Hkd63(1)[AB641940]	3			3						4	0
Hkd64	Lake Rausu-ichinonnuma, Hokkaido	44.0297˚N	145.1042˚E	695	absent	*D. dentifera*	2	Hkd64(2)[AB642019]	0			3			1	2					8	3
Hkd65	Lake Rausu-sannnonuma, Hokkaido	44.0319˚N	145.1006˚E	705	absent	*D. dentifera*	3	Hkd65(3)[AB642019]	1		Hkd65(1)[AB641941]	3			3						6	3
Hkd66	Lake Rausu-gonouma, Hokkaido	44.0325˚N	145.0869˚E	760	absent	*D. dentifera*	2	Hkd66(2)[AB642020]	1		Hkd66(1)[AB641942]	3			3						2	0

(B)

Species	ID	Location	Accession no.	
			12S	ITS
D.dentifera	USA, AK1a	Conners Lake, AK; USA	AY730373	AY730384
D.dentifera	USA, AK1b	Conners Lake, AK; USA	AY730372	
D.dentifera	USA, AK2a	Teller6 Pond, AK; USA		AY730383
D.dentifera	USA, AK2b	Teller6 Pond, AK; USA	AY730374	
D.dentifera	USA, IN1	Hammond Lake, IN; USA		AY730389
D.dentifera	USA, IN2	Wylund Lake, IN; USA		AY730387
D.dentifera	USA, NYa	Deep Lake, NY; USA		AY730390
D.dentifera	USA, NYb	Deep Lake, NY; USA	AY730375	
D.dentifera	USA, WI	Mud Lake, WI; USA	AY730376	AY730391
D.galeata	SCO	Loch Oich; Scotland	AY730362	
D.galeata	USA, AK1	Alder Pond, AK; USA	AY730363	AY730378
D.galeata	USA, AK2	Mirror Lake, AK; USA	AY730365	AY730397
D.galeata	USA, AK3	Summit Lake, AK; USA	AY730367	AY730396
D.galeata	USA, AK4	Weiner Lake, AK; USA	AY730366	AY730379
D. galeata	USA, IN	James Lake, IN; USA		AY730380
D.galeata	USA, CA	Los Carneros, CA; USA	AY730368	AY730381
D.galeata	CZE	Slapy Reservior; Czech Republic	U34647	
D.galeata	ENG	Ullswater; England	AY730360	
D.galeata	DEU	Bodensee; Germany	AY730361	AY730399
D.galeata	USA, MI	Baseline Lake, MI; USA	AY730369	AY730388
D.galeata	CAN, NB	Loch Lomond, NB; Canada	AY730364	AY730382
D.galeata	USA, OR	Lost Lake, OR; USA	U34650	
D.longispina	POL	Nizny Toporowy Staw (Pond); Poland	U34638	
D.rosea	SLO	Rohacske pleso Dolni; Slovakia	AY730371	
D.rosea	CHE	Arosa; Switzerland	AY730370	
D.cucullata	DEU	Schierensee; Germany	U34652	
D.cucullata	NLD	Tjeukemeer; the Netherlands	AF277271	
D.umbra	NOR	Jotunheimen; Norway	AF277276	
