# Supplementary figures and images for: The long-term consequences of hybridization between the two Daphnia species, D. galeata and D. dentifera, in mature habitats
Source: BMC Evol Biol. 2011 Jul 15;11:209. doi: 10.1186/1471-2148-11-209 (PMC3156774; doi:10.1186/1471-2148-11-209)

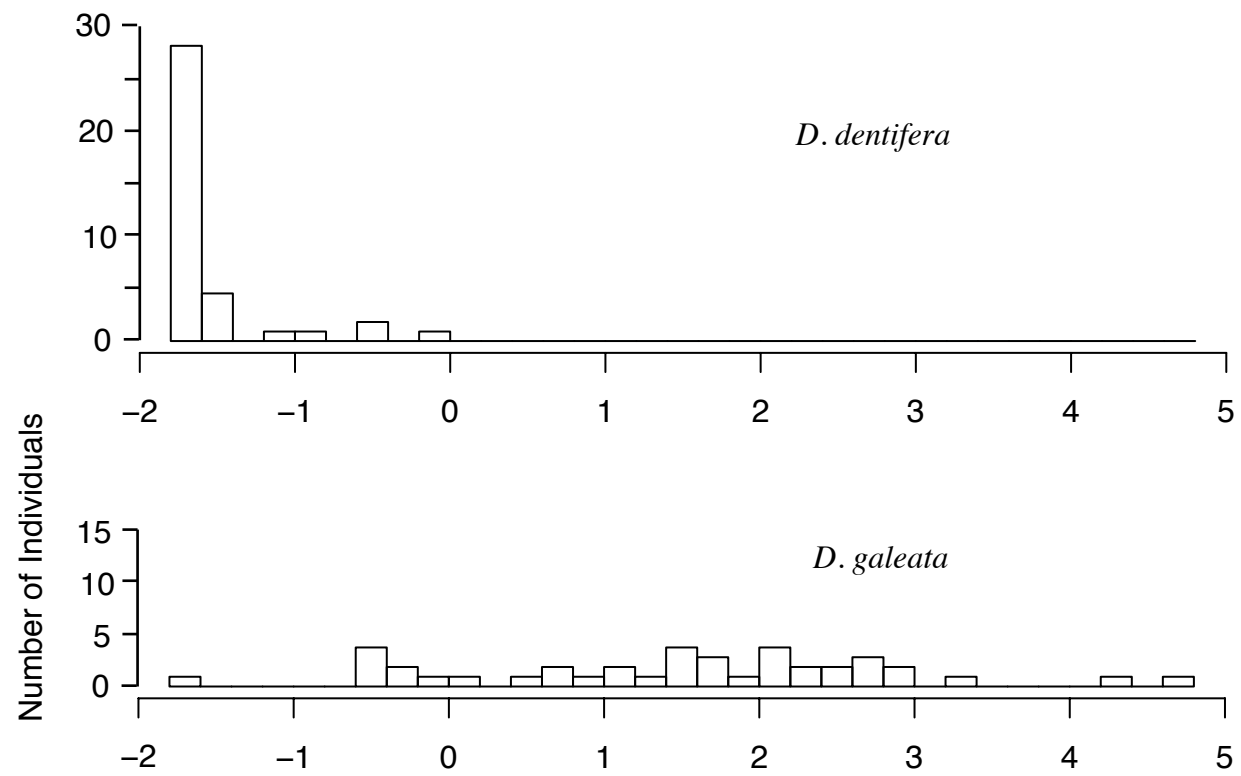

Supplement: Additional file 2 — Frequency distribution of individuals of the scores of linear disciminators for mitochondrial D. dentifera (above panel) and D. galeata (below panel). Upper panel shows the frequency distribution of the specimens with D. dentifera mtDNA, and lower panel shows that of the specimens with D. galeata mtDNA. [file 1471-2148-11-209-S2.PDF]

(A)

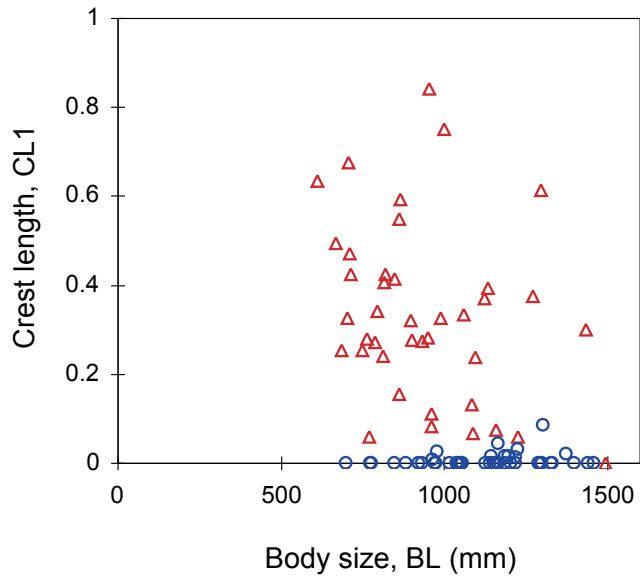

(B)

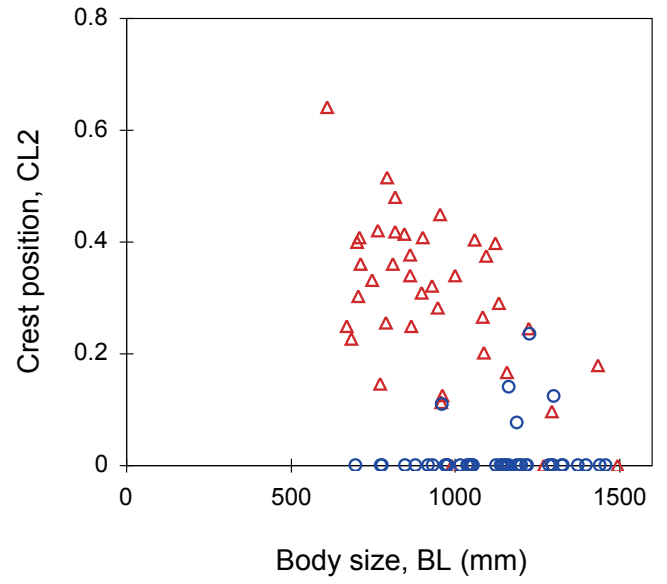

Supplement: Additional file 3 — Relationship between body size (BL), relative crest length (CL1), and relative crest position (CL1). (A) A plot of the relationship between BL and CL2. (B) A plot of the relationship between BL and CL1. Red triangles and blue circles represent specimens with D. galeata mtDNA and those with D. dentifera mtDNA, respectively. [file 1471-2148-11-209-S3.PDF]

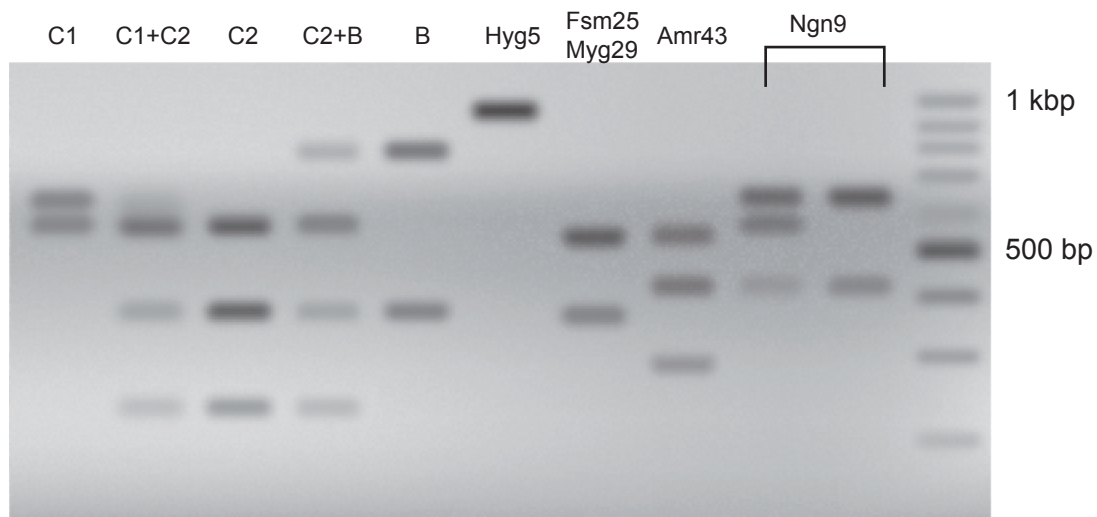

Supplement: Additional file 4 — An image of an agarose gel with the five main patterns (C1, C1+C2, C2, C2+B, and B) and the five minor patterns of specimens with D. galeata mtDNA in Hyg5, Fsm25, and Myg29, and specimens with D. dentifera mtDNA in Amr43 and Ngn9. The patterns were obtained by digesting the nuclear ITS-2 region with the RsaI restriction enzyme. The last lane is a size ladder with rungs from 300 bp to 1000 bp. [file 1471-2148-11-209-S4.PDF]

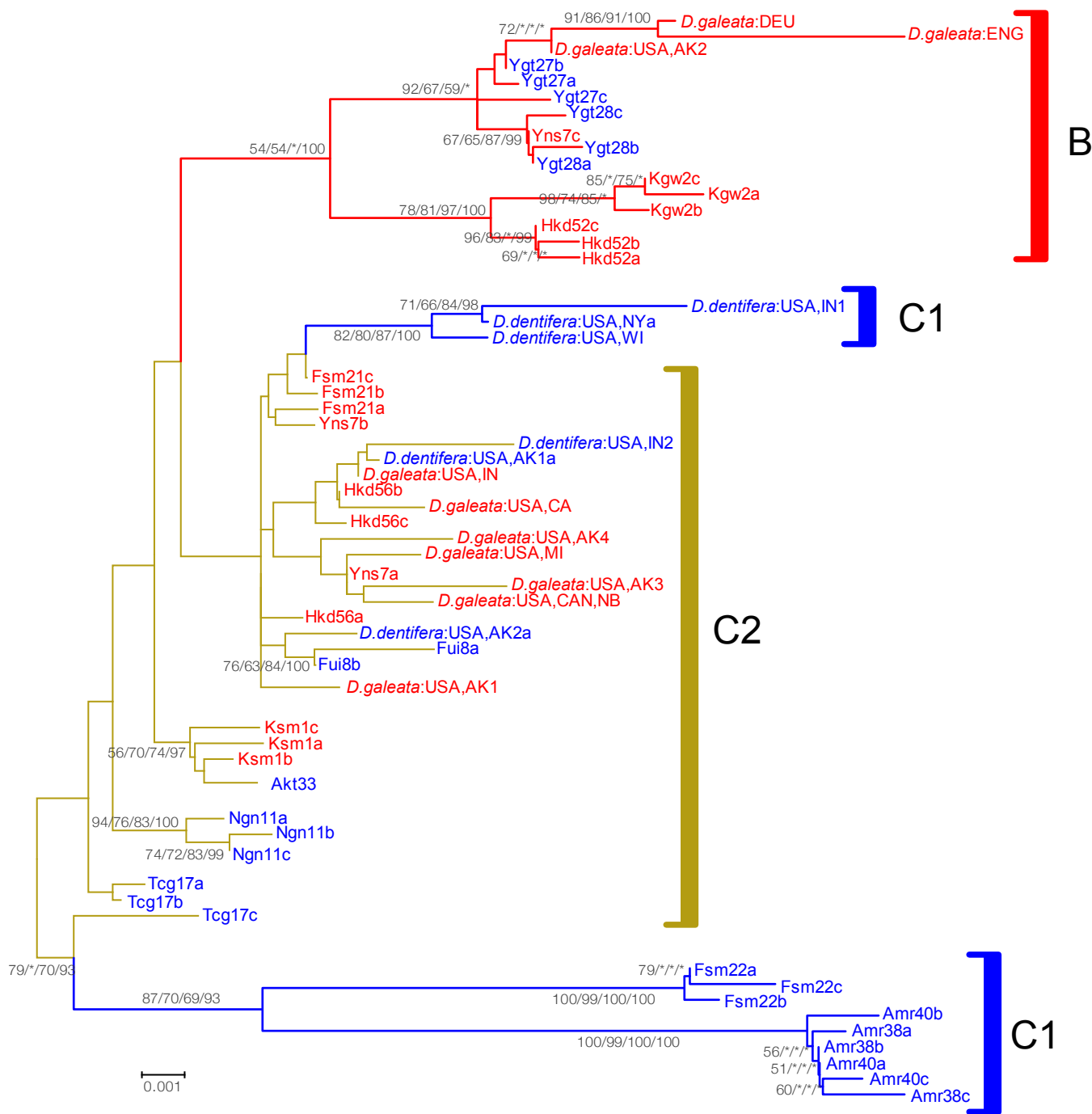

Supplement: Additional file 5 — Neighbor-joining (NJ) phylogram of the nuclear ITS-2. The four numbers on each branch indicate greater than 50% bootstrap support values for the branch as determined by NJ, maximum parsimony, and maximum likelihood methods, and a Bayesian clade credibility value of greater than 70%. Asterisks indicate values with less than 50% bootstrap support values, less than 70% Bayesian support values, or no support. We distinguished the color of the branch line based on the ITS-1 RFLP patterns (B, C1, and C2). We used a red line for the sequences of pattern B, a blue line for those of pattern C1, a gold line for those of pattern C2, red characters for the specimens with D. galeata mtDNA, and blue characters for those with D. dentifera mtDNA. [file 1471-2148-11-209-S5.PDF]
